# Advancing Sterilization of Medical Implant Polymers: Novel Low-Temperature Deep-vacuum Vaporized H_2_O_2_ Technology Surpasses Current Methods

**DOI:** 10.1007/s11095-026-04063-x

**Published:** 2026-03-16

**Authors:** Abinaya Nallathambi, Enrica Chiesa, Mariella Rosalia, Giovanna Bruni, Aurora Tamborini, Sergio Crotti, Ida Genta

**Affiliations:** 1 Department of Drug Sciences, University of Pavia, 27100 Pavia, Italy; 2 Department of Physical Chemistry, University of Pavia, 27100 Pavia, Italy; 3 De Lama S.p.A., 27028 S. Martino Siccomario (Pavia), Italy

**Keywords:** electrospun scaffolds, low-temperature sterilization, vaporized hydrogen peroxide sterilization

## Abstract

**Purpose:**

To develop a deep vacuum vaporized hydrogen peroxide (VH₂O₂) sterilization method capable of preserving the structural and functional integrity of biocompatible polymeric electrospun mats usable as advanced biocompatible medical implants and devices, with particular focus on the influence of vacuum level during treatment.

**Methodology:**

Electrospun mats made of poly-L-lactide-co-glycolide (PLGA), poly-L-lactide-co-ε-caprolactone (PLC), and thermoplastic polyurethane (TPU) were prepared and sterilized with VH₂O₂ under low-vacuum (LV, 10 mbar) and high-vacuum (HV, 2 mbar) conditions at 20–50 °C. Scanning electron microscopy (SEM) assessed fiber morphology and pore area, contact angle measurements evaluated surface wettability, Fourier-transform infrared spectroscopy (FTIR) detected chemical modifications, and gel permeation chromatography (GPC) analyzed weight-average molecular weight (Mw) and polydispersity index (PI).

**Results:**

HV sterilization caused fiber compression across all polymers, increasing fiber diameter and reducing pore area. LV sterilization conditions prevented morphological changes in TPU and PLC, while PLGA remained sensitive under both vacuum levels. PLGA also exhibited increased hydrophilicity due to surface reorganization and transition from Cassie–Baxter to Wenzel-type wetting. FTIR showed no chemical changes, and GPC confirmed stable Mw and PI for all tested materials (PLGA: 25–27 kDa; PLC: 16–18 kDa; TPU: 23–25 kDa).

**Conclusion:**

Deep vacuum VH₂O₂ sterilization at room temperature is feasible, with vacuum level critical for maintaining scaffold integrity. LV sterilization effectively preserves morphology in PLC and TPU, while HV is also reliable for less sensitive polymers. Chemical and molecular properties remain unaffected, supporting VH₂O₂ sterilization as a gentle and effective promising method for implantable textile-based biomaterials.

**Supplementary Information:**

The online version contains supplementary material available at 10.1007/s11095-026-04063-x.

## Introduction

Recent advancements in material science have introduced a variety of low thermal resistant polymers and soft materials, including hydrogels, water-based materials majorly used in drug delivery; and highly sensitive to temperature, and shape-memory polymers, which can return to a pre-defined shape upon exposure to a stimulus (e.g., temperature change); and thermoplastic elastomers (TPEs) widely used in medical devices due to their flexibility and elasticity. These materials are engineered for enhanced biological compatibility and specific functional properties, but they are often incompatible with conventional sterilization methods that rely on high temperatures or harsh chemical conditions Most of these applications need the sterilization of the final drug delivery system or medical device. Classical moist–heat sterilization (steam autoclaving) and dry-heat sterilization are high-temperature methods that remain the gold standard when materials can withstand both heat and humidity. Moist heat sterilization (ISO 17665) uses saturated steam (commonly 121 °C for 15–30 min or 134 °C shorter cycles) to inactivate microorganisms by coagulation/denaturation of proteins; it provides excellent penetration for porous loads but is incompatible with hydrolytically-sensitive polymers (e.g., aliphatic polyesters such as poly-lactide, PLA, poly-lactide-co-glycolide, PLGA, and other related derivatives), which undergo hydrolytic chain scission, dimensional change and loss of mechanical integrity when exposed to steam and elevated temperature [1, 2]. Dry heat sterilization (ISO 20857), typically performed at 160–180 °C for prolonged periods, achieves microbial inactivation primarily through oxidative and dehydration effects [3]. While effective for materials that tolerate heat but not moisture, it is rarely suitable for most biomedical polymers used in soft tissue scaffolds, particularly thermolabile electrospun structures due to risks of thermal degradation, unless the polymer has demonstrated heat resistance and the process has been validated for the specific material (ISO/TS 16775:2021) [4]. Ethylene oxide (EtO) sterilization is one of the most established low-temperature techniques, operating at 37–63 °C and capable of penetrating complex geometries, making it suitable for heat-sensitive and moisture-sensitive polymers. however, it requires prolonged aeration to remove toxic residues [4, 5].

Literature shows traditional autoclaving sterilization compromised the shape-memory properties and overall structural integrity [6], and TPEs widely used in medical devices due to their flexibility and elasticity can undergo degradation or loss of mechanical properties when subjected to high-temperature sterilization techniques [7]. Moreover, growing concerns from regulatory bodies regarding EtO’s carcinogenic potential and environmental impact have necessitated the development of safer alternative sterilization techniques [4]. Consequently, new sterilization methods for medical devices and polymeric biomaterials are under continuous development, each offering distinct mechanisms of microbial inactivation and specific compatibility profiles with different material classes.

Gamma irradiation is widely used in single-use medical devices and ensures high microbial inactivation via ionizing radiation, but its high penetration depth can lead to polymer chain scission, oxidation, and changes in crystallinity, especially in polyesters and tyrosine-derived polycarbonates [5, 8]. Electron beam sterilization operates on the same principle as gamma irradiation but with shallower penetration, enabling precise dose control and shorter processing times, although it is less suitable for bulky or dense products [8]. Other chemical sterilants such as ozone gas and peracetic acid have been investigated for temperature-sensitive devices; ozone provides oxidative microbial killing without high heat, and peracetic acid offers broad-spectrum antimicrobial efficacy with minimal thermal load, though both require compatibility assessments for potential oxidative damage to polymers [5, 8]. In January 2024, the U.S. Food and Drug Administration (FDA) formally recognized vaporized hydrogen peroxide (VH₂O₂) as an Established Category A sterilization method for medical devices, citing its long-standing clinical use, proven effectiveness, and lower toxicity compared to EtO, which is increasingly scrutinized for its carcinogenic risks and environmental impact [9]. Likewise, the European Commission, through Regulation (EU) 2022/1423, authorized hydrogen peroxide (H₂O₂) for sterilization under the Biocidal Products Regulation, reinforcing its acceptance as a safe and effective option in medical device manufacturing workflows [10]. Hence, this study investigates the effectiveness of the application of low-temperature VH₂O₂ sterilization on the three medical-grade polymers, largely used in the preparation of medical devices/drug delivery systems, specifically PLGA, poly L-lactide and ε-caprolactone (PLC), and thermoplastic polyurethane (TPU). In particular, these materials were chosen due to their potential suitability for the fabrication of synthetic vascular and cardiac sustitutes. PLGA and PLC were selected because they are appropriate for resorbable, non-load-bearing vascular graft layers that rely on controlled, sequential degradation to support staged tissue regeneration and drug release [11, 12]. In contrast, biocompatible TPU serves as the non-degradable structural layer, providing the long-term mechanical strength and compliance required to match native vascular tissue [13].

The study subjected the polymers and the related electrospun mats, selected as representative models for prosthetic implant designs, to various low-temperature VH₂O₂ sterilization protocols, following ISO/TS 16775 and ISO 22441:2022 guidelines [4, 14]. Sterility was verified using *Geobacillus stearothermophilus* in accordance with ISO 11737–2 [15]. The process was fine-tuned to identify conditions that minimized raw material alterations and electrospun mats as well, resulting in an optimized protocol. The impact of sterilization on chemical, thermal, mechanical, and morphological properties was comprehensively assessed using gel permeation chromatography, Fourier-transform infrared spectroscopy, differential scanning calorimetry, surface wettability analysis, tensile testing, and scanning electron microscopy. Focusing on sterilization-induced physicochemical changes, this approach supports the safe and effective use of implantable polymers by identifing a sterilization method that preserves material performance and reliability, thereby providing a solid basis for subsequent biological validation.

## Materials and Methods

### Materials

Poly (L-lactide-co-glycolide) (PLGA, PURASORB PLG 8531, 85:15 molar ratio of lactide to glycolide) and poly (L-lactide-co-ε-caprolactone) (PLC, PURASORB PLC 7015, 70:30 molar ratio) were obtained from IMCD (Köln, Germany). Thermoplastic polyurethane (TPU, Pellethane®, 2363-80AE) was supplied by Lubrizol (Ohio, USA). Solvents used included 1,1,1,3,3,3-hexafluoro-2-isopropanol (HFIP, ≥ 99%, Fluorochem Ltd, Glossop, UK), dichloromethane (DCM; analytical grade), tetrahydrofuran (THF, analytical grade), and chloroform (analytical grade) were from Carlo Erba Reagents (Milan, Italy). Dulbecco’s phosphate-buffered saline (PBS, 10 ×, sterile) was purchased from Carlo Erba Reagents (Milan, Italy). Tryptic Soy Broth (TSB) was used for culturing biological indicators and sourced from a standard microbiological media supplier. All other reagents used were of analytical grade.

### Methods

#### Electrospinning of Fibres

Solutions for electrospinning were prepared as follows: 10% w/v PLGA in HFIP, 5% w/v TPU in HFIP, and 20% w/v PLC in DCM. Electrospinning was performed using a NANON 01 A vertical electrospinning device (MECC Co., Fukuoka, Japan) through a 22-gauge needle at a feed rate of 0.6 mL/h, needle-to-collector distance of 15 cm, and an applied voltage of 20 kV. The process was carried out under controlled environmental conditions (22 ± 2 °C and 40 ± 5% relative humidity) using a stationary flat collector. Electrospinning was performed for 30 min per mat; for each polymer, three mats were produced in a single batch. Each mat was divided into three matched portions for reference non-sterilized (NS), low-vacuum (LV) and high-vacuum (HV) VH₂O₂ sterilized specimens, packed in Tyvek bag, and stored in a desiccator at room temperature until further use, enabling paired comparisons across all analyses.

#### Sterilization

The raw polymer materials and relative electrospun mats were sterilized using a novel VH₂O₂ system (HyPerPure®, De Lama S.p.A., Pavia, Italy). The system incorporates the patented HyPerGeneSys® generator, able to operate in a high vacuum (< 1 mbar) and in addition by acting with an electromagnetic field capable of ionizing the peroxide molecules in order to increase their lethal effect on microorganisms. This system operates under high vacuum and low-oxygen conditions, delivering a precise dose of 8 g of 35% w/w H₂O₂ into the sterilization chamber. The sterilization cycle consists of vacuum conditioning, chamber gassing with VH₂O₂, a 15 min decontamination period at the selected chamber pressure, followed by gradual pressure release and aeration. During aeration, residual H₂O₂ is catalytically decomposed into water and oxygen, ensuring the process is environmentally sustainable and free of toxic residues. The total process duration was 40–45 min.

a Set-up of Sterilization Conditions
  Sterilization parameters were first optimized using raw polymer pellets at varying temperatures (in the range 20–50 °C) under a fixed vacuum condition (2 mbar). Raw materials were weighed and placed in sterilizable Tyvek bags suitable for sterilization. The temperature and vacuum conditions tested for both raw materials and electrospun scaffolds are summarized in Table I.

**Table I:** Sterilization conditions tested for raw materials and electrospun scaffolds

Material	Raw Material		Electrospun Scaffold	
	Temperature (°C)	Vacuum (mbar)	Temperature (°C)	Vacuum (mbar)
PLGA	30, 40, 50	2	30	2, 10
PLC	20, 30	2	20–25(r.t.)	2, 10
TPU	30, 40, 50	2	30	2, 10

*r.t.* room temperature.
  Gel permeation chromatography (GPC) was performed to evaluate the effect of temperature (30, 40, 50 °C) on the polymer molecular weight.
  The selected temperatures for each polymer were then applied to the related electrospun scaffolds, which were tested under varying vacuum conditions, namely low-vacuum (10 mbar, LV) and high-vacuum (2 mbar, HV) (Table I). Electrospun scaffolds were cut into specimens suitable for mechanical testing according to ASTM D882 and packaged in sterilizable Tyvek bags before sterilization.
  Sterility was confirmed using biological (BIs) and chemical (CIs) indicators, and samples stability was assessed using proper physico-chemical characterization.
b Sterilization Validation: Chemical and Biological Indicator Assessment
  Sterility was assessed using CIs and BIs. H₂O₂-sensitive strips (Cross Checks P, SteriTec, Athens, Texas, USA) confirmed sterilant contact by color change. Spore strips (BIONOVA®, Resim BT92/6) containing *Geobacillus stearothermophilus* (10⁶ CFU/strip) were used as biological indicators, compliant with IRAM 37102–1 and ISO 11138–1 [15, 16]. Samples were packaged in Tyvek® bags.
  For each condition, 3–5 BIs were tested in at least two independent trials. After sterilization, BIs were incubated in Tryptic Soy Broth at 58 °C for 24 h. Sterility was confirmed by absence of turbidity, while positive controls showed growth. A Sterility Assurance Level (SAL) of 10⁻⁶ was considered achieved when treated BIs showed no growth and controls were positive.

#### Physico-chemical Characterization of Raw Materials and Electrospun Scaffolds

a Gel permeation Chromatography (GPC)
  Molecular weight parameters and polydispersity index (PI) were determined using an Agilent 1260 Infinity system (Cernusco sul Naviglio — MI, Italy) with three Pheno GEL columns (5 µm, pore sizes 500 Å, 10^3^ Å, 10^4^ Å) and THF as mobile phase (1 mL/min). Raw materials were analyzed as such and after sterilization at 30, 40, and 50 °C (PLGA, TPU) and 20, 30 °C (PLC) (Table I). Electrospun scaffolds were analyzed after sterilization at 30 °C (PLGA, TPU) and 20–25 °C (PLC) under high-vacuum conditions (HV, 2 mbar). PLGA samples were dissolved in chloroform, TPU and PLC in THF (1 mg/mL). Injection volume: 20 µL. Mn, Mw, and PDI were calculated using a polystyrene calibration curve (Mw: 1,480–361,500 Da). Results: mean ± SD (*n* = 3).
b Differential Scanning Calorimetry (DSC)
  The thermal properties of PLGA, PLC, and TPU, as raw materials and electrospun scaffolds, were analyzed before and after sterilization under the conditions listed in Table I, specifically under HV and low-vacuum (LV) conditions (10 mbar). Measurements were performed using a DSC Q2000 (TA Instruments, New Castle, DE, USA) under nitrogen flow (50 mL/min). Samples (~ 3 mg) were hermetically sealed in aluminium pans.
  For PLGA and PLC, a first cycle from 0–70 °C at 2 °C/min erased thermal history, followed by cooling to 0 °C at 2 °C/min to allow controlled recrystallization. A second cycle from 0–180 °C at 2 °C/min was used for thermal property analysis. TPU, with T_g_ ~ –60 °C and negligible cold crystallization, was analysed using a single heating cycle from 0–180 °C at 2 °C/min.
c Surface wettability analyses
  Static contact angle measurements of electrospun PLGA, PLC, and TPU mats, non-sterilized and sterilized under Table 1 conditions (LV—HV), were performed using a Contact Angle Meter DMe-211 (Kyowa Interface Science Co., Ltd., San Prospero - MO, Italy). Mats (2 × 2 cm) were mounted with adhesive tape; 5 µL bi-distilled water droplets were deposited. Images captured and angles calculated using analysis software FAMAS v3.3. Measurements performed at 22–25 °C, three replicates per sample; results mean ± SD. To evaluate whether sterilization conditions produced significant differences in contact angle for each polymer, data were tested for normality and analyzed using one-way ANOVA for normally distributed datasets and the Kruskal–Wallis test for non-parametric distributions.
d Attenuated Total Reflectance Fourier-Transform Infrared Spectroscopy (ATR-FTIR)
  ATR-FTIR spectra of electrospun PLGA, PLCL, and TPU mats, before and after sterilization under varied vacuum conditions (LV—HV), were acquired using a Nicolet FT-IR iS20 (Nicolet, Madison, WI, USA) with a Smart iTR diamond ATR. Spectra (4000–500 cm⁻^1^, 4 cm⁻^1^ resolution, 32 scans) were collected directly on mats. Characteristic bands for urethane, ester, and ether bonds were analysed to assess potential chemical changes due to sterilization.

#### Morphological Characterization of Electrospun Scaffolds

Surface morphology of electrospun PLGA, PLCL, and TPU mats, non-sterilized and sterilized under varied vacuum conditions (LV—HV), were evaluated by SEM (Zeiss EVO MA 10, Carl Zeiss, Germany). Mats (~ 0.5 cm^2^) were mounted on aluminium stubs with carbon tape and sputter-coated with gold. Fiber diameters were measured using ImageJ (v1.54w) with the Diameter J plugin; at least 50 fibers per sample were analyzed. Results are reported as mean ± SD.

#### Mechanical Characterisation of Electrospun Scaffolds

Tensile properties of electrospun PLGA, PLC, and TPU mats, cut into specimens suitable for mechanical testing according to ASTM D882, sterilized and non-sterilized under Table I conditions, were measured using a motorized tension/compression Test Stand ESM303 (Mark-10, Copiague, NY, USA) equipped with a 25 N load cell and MESUR® Gauge Plus software. Dog bone-shaped specimens (80 × 10 × 4 mm) were prepared using a die cutter according to ASTM D882. Tests were performed before and after sterilization.

Tensile tests were conducted at a constant extension rate of 1.4 mm/min. Load (N) versus displacement (mm) data were recorded and subsequently converted to stress (MPa) versus strain (mm/mm) curves using the following equations:

1 $$E=\frac{\Delta \sigma }{\Delta \varepsilon }$$

where ∆σ is the change in stress in the linear elastic region, and ∆ε is the corresponding change in strain.

2 $$\text{Elongation at break }(\mathrm{\%}) =\left(\frac{{L}_{f}-{L}_{0}}{{L}_{0}}\right)\times 100$$

where L_0_​ is the original gauge length and L_f​_ is the final length at break;

3 $${\sigma }_{UTS} =\frac{{F}_{max}}{A}$$

where F_max_​ is the maximum load before fracture and A is the original cross-sectional area of the sample.

4 $${\sigma }_{y}=\frac{{F}_{y}}{A}$$

where ${\sigma }_{y}$​ is the load at the yield point, F_y_​ is defined as the stress at which permanent deformation begins, and A is the original cross-sectional area.

The Young’s modulus and elongation-at-break (breaking point), ${\sigma }_{y}$, ${\sigma }_{UTS}$, were determined from stress vs. strain curves, and the results were expressed as average ± standard deviation (*n* = 3). Statistical analysis was performed using the paired Student’s *t*-test. Pairwise comparisons were made between the non-sterilized (NS) group and each sterilization condition, LV and HV, to independently assess the effect of each treatment.

## Results and Discussion

To establish an effective sterilization protocol, preliminary DSC characterization was performed on PLGA, PLC, and TPU raw polymers to determine their glass transition temperatures (T_g_). This was critical, as sterilization at or above T_g_ could increase polymer chain mobility or promote degradation, potentially causing fiber fusion, deformation, and loss of mechanical integrity in final electrospun scaffolds [17, 18]. Based on these characterization results (*unreported data*), four sterilization temperatures (20, 30, 40, and 50 °C) were selected, all below or near the polymers T_g_. Notably, PLC exhibited a T_g_ of ~ 10–20 °C, highlighting its sensitivity even to mild thermal exposure and the need for careful temperature control.

Sterilization of raw polymers was then carried out using a VH₂O₂ system (HyPerPure®, De Lama, Pavia, Italy) in thermal and pressure conditions as reported in Table I.

Polymers stability was assessed by DSC and GPC. Comparison of non-sterilized and sterilized TPU, PLC, and PLGA raw polymers showed no changes in molecular weight distribution (Mn, Mw, PDI) or thermal properties (*see* Supplementary data, Fig. S1,a and S2), confirming that the selected conditions preserved structural and molecular integrity. Sterility was confirmed using CIs and BIs. Color changes in the CIs and the absence of microbial growth in the BIs (Fig. S3) demonstrated complete sterilization of all samples under the tested temperature conditions in the HV setup. To establish a safe and effective sterilization method and able to preserve morphological and mechanical characteristics as well of the electrospun mats, the mildest temperature conditions were selected: 20–25 °C for PCL and 30 °C for PLGA and TPU respectively.

The optimized protocols were then applied to electrospun TPU, PLGA, and PLC scaffolds and testing LV and HV conditions (Table I). Subsequent analyses included molecular characterization (GPC, DSC), morphology (SEM), surface wettability (contact angle), structural evaluation (FTIR), and mechanical testing under varying vacuum conditions at constant sterilization temperature.

### Electrospun scaffolds Sterilization Assessment

Sterility validation was conducted in accordance with ISO 11737–2:2019 [19] which outlines microbiological methods for defining, validating, and maintaining sterilization processes for healthcare products. BIs containing *Geobacillus stearothermophilus* spores were used to evaluate sterilization efficacy for raw polymers (PLGA, PLC, TPU) and the related electrospun scaffolds under all tested conditions. Sterilization was performed at 30 °C for all polymers and at room temperature (20–25 °C) for PLC, under two vacuum conditions: HV (2 mbar) and LV (10 mbar). BI codes were assigned to track each condition: HV (30 °C) – PLC: BI 01(F), TPU: BI 02(F); LV (30 °C) – PLGA: BI 01, PLC: BI 0, TPU: BI 03; room temperature – PLC: BI 01 at HV, BI 02 at LV.

**Fig. 1:**
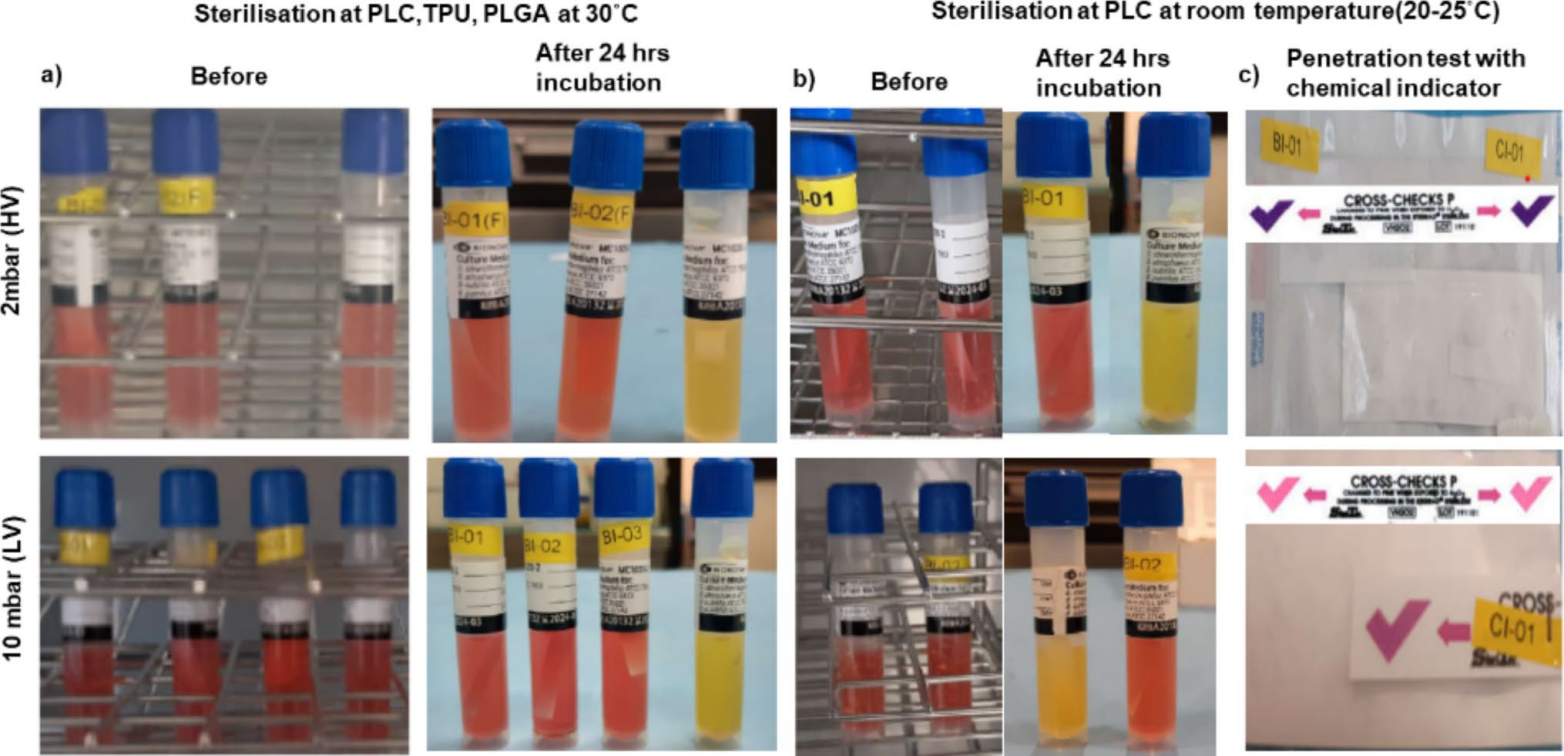
Sterility assessment of electrospun scaffolds using BIs and CIs (representative samples). (**a**) BIs for PLC, TPU, and PLGA scaffolds under HV and LV conditions at 30 °C, (**b**) PLC at room temperature (20–25 °C). Red color after 24 h incubation indicates complete spore inactivation, while positive controls turned yellow, confirming spore viability. (**c**) CIs showing color change from purple to pink upon exposure to vaporized H₂O₂, confirming effective sterilant penetration and surface contact. Representative results are shown for PLC at room temperature; all scaffolds exhibited consistent CI responses under both HV and LV conditions.

Following sterilization, BIs were incubated at 58 °C for 24 h and monitored for color change. In all sterilized samples, BIs retained their original red color, indicating complete inactivation of spores and absence of bacterial growth, while positive controls (unsterilized BIs) turned yellow and became turbid, confirming spore viability and validating the testing system (Fig. 1a,b). These results demonstrate that the sterilization process achieved a sterility assurance level (SAL) of 10⁻⁶ across all polymers, temperatures, and vacuum conditions.

VH₂O₂ penetration into sealed scaffold packages was verified using Cross-Check P chemical indicators (CIs) in accordance with ISO 11140–1:2014 [20]. CI strips consistently exhibited a color change from purple to pink, confirming effective sterilant diffusion and surface contact throughout the packaged scaffolds (Fig. 1c). A representative CI result for PLC at room temperature is shown; all scaffolds, including TPU and PLGA, displayed consistent CI color change and complete BI inactivation under both HV and LV conditions.

No differences in sterilization efficacy or sterility retention were observed between HV and LV or across temperature ranges, including room-temperature treatments. These findings confirm the robustness, reproducibility, and thermal compatibility of VH₂O₂ sterilization, even under mild conditions suitable for thermally sensitive biomaterials.

## Electrospun Scaffolds Analysis Pre- and Post-Sterilization

### Molecular Weight Analysis

**Fig. 2:**
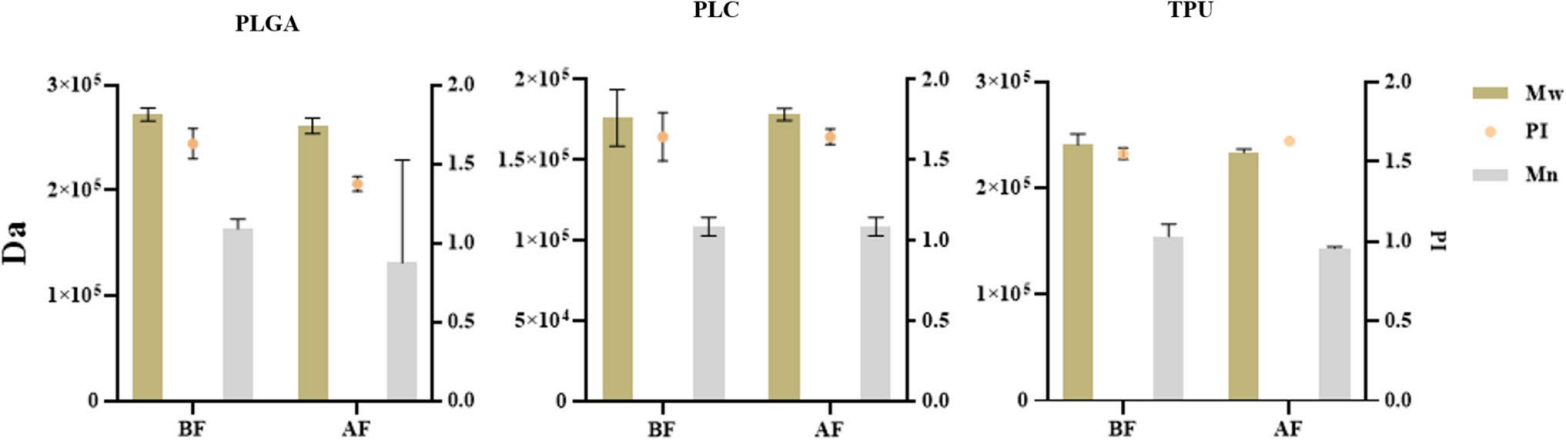
Comparative analysis of number-average molecular weight (M_n_), weight-average molecular weight (M_w_), and polydispersity index (PI) of electrospun PLGA, PLC, and TPU scaffolds before (BF) and after (AF) VH₂O₂ sterilization. Sterilization was performed at 20 °C for PLC and 30 °C for PLGA and TPU under 2 mbar vacuum conditions. Paired *t*-test (*n* = 3) revealed no significant differences (*p* > 0.05), indicating preservation of molecular integrity.

Molecular weight analysis was performed to assess whether VH₂O₂ sterilization alters the polymer chain integrity of electrospun scaffolds. GPC analysis showed no statistically significant differences in Mn, Mw, PI between electropsun samples before and after sterilization for PLGA, PLC, or TPU (paired t-test, *n* = 3, p > 0.05) (Fig. 2 – *see* related chromathograms in Supplementary Fig. S1,b). Mw values remained stable within narrow ranges following sterilization, measuring 25–27 kDa for PLGA, 16–18 kDa for PLC, and 23–25 kDa for TPU.

The preservation of Mw and PI indicates that VH₂O₂ sterilization under low-temperature conditions did not induce detectable chain scission or crosslinking, despite the known susceptibility of polymers to degradation during sterilization processes involving reactive species or radiation, due to conventional sterilization methods such as gamma irradiation [2, 5].

### Thermal Analysis

**Fig. 3:**
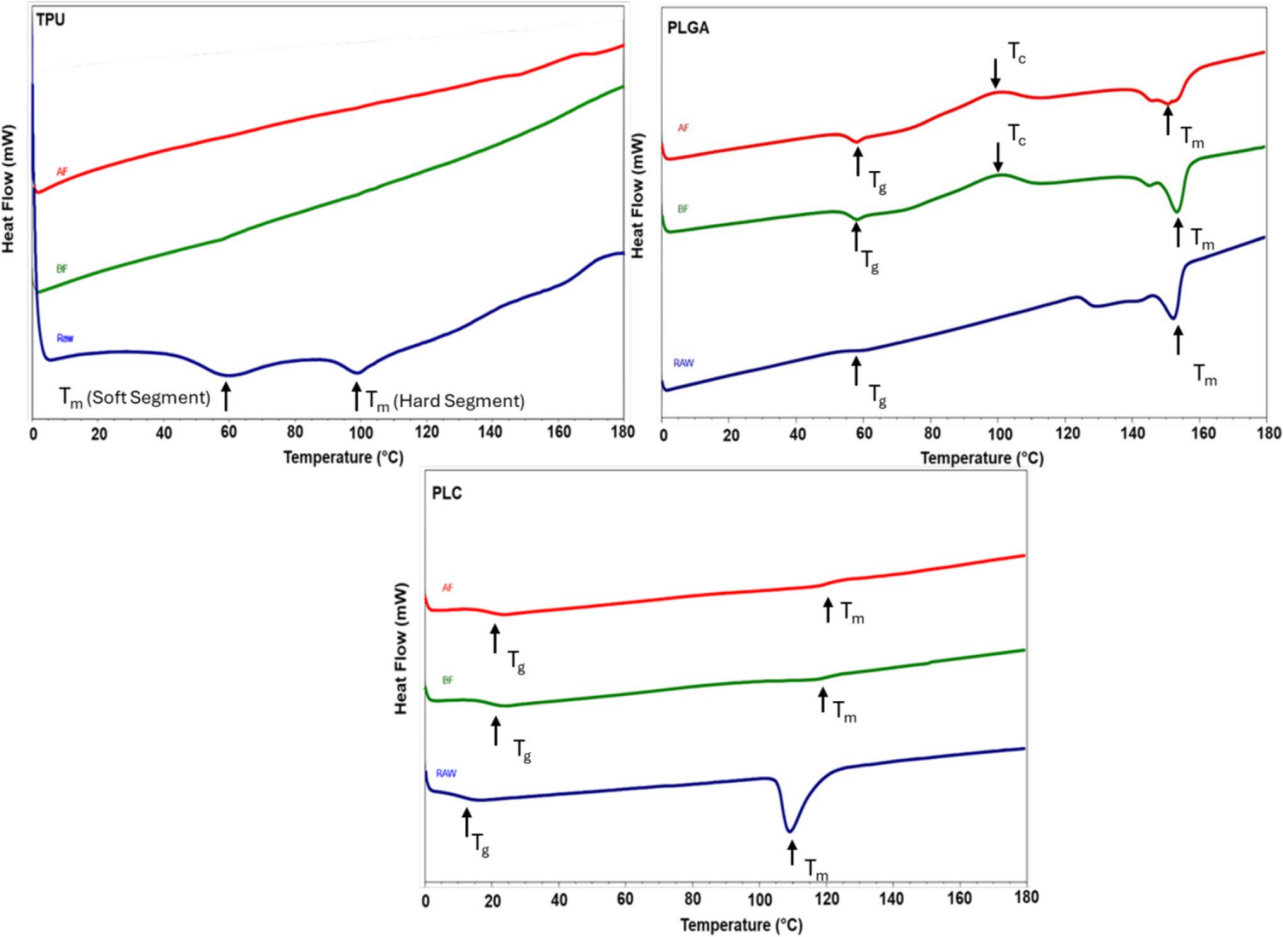
Differential Scanning Calorimetry (DSC) thermograms of TPU, PLGA and PLC in their electrospun form, shown before (BF) and after (AF) sterilization, alongside the corresponding raw materials for comparison.

Thermal analysis was performed to assess the impact of electrospinning and VH₂O₂ sterilization on the thermal behavior of the polymer scaffolds. DSC thermograms obtained from the second heating cycle showed a marked reduction in heat flow for all electrospun mats compared to their corresponding raw materials (Fig. 3).

Following sterilization, PLGA and PLC exhibited a slight increase in enthalpy, whereas TPU displayed a distinct thermal response.

TPU raw material exhibited complex thermal transitions, including endothermic peaks associated with short- and long-range order [21]. In contrast, electrospun TPU mats showed disappearance of a characteristic endothermic peak, and no distinct T_g_ or T_m_ was detected. FTIR analysis confirmed that no changes in chemical structure occurred following electrospinning (*see* Supplementary Fig. S4), indicating that the observed thermal changes are attributable to altered morphological organization rather than chemical degradation [22].

For PLGA (85:15), the melting enthalpy (ΔH) decreased from 32.42 J/g in the raw material to 14.44 J/g after electrospinning. Electrospun PLGA mats exhibited a crystallization peak that was not observed in the raw material. Sterilization did not significantly affect the melting temperature (T_m_: 147.82 °C before sterilization *vs* 147.52 °C after sterilization) or glass transition temperature (T_g_: 56.67 °C *vs* 56.44 °C). A modest increase in melting enthalpy (ΔH: 14.44 to 18.24 J/g) and a slight decrease in crystallization temperature (T_c_: 87.18 °C to 82.32 °C) was observed after sterilization. As the enthalpy of fusion for fully crystalline PLGA is undefined, crystallinity values normalized to PLA (93 J/g) are reported as relative indicators of structural ordering [17, 18]. The crystallization peak observed in the electrospun mats is therefore attributed to recrystallization during the DSC thermal cycle rather than to intrinsic structural changes induced by electrospinning or sterilization.

For PLC (70:30), electrospinning resulted in a broad and diffuse melting endotherm compared to the sharp transition observed in the raw pellets, preventing reliable determination of T_m_. Crystallinity decreased from approximately 12% in the raw material to ~ 0.5% in the electrospun scaffold. A shift in T_g_ from ~ 10 °C in the raw material to ~ 20 °C in the scaffold was observed. The thermal behavior of PLC is known to be strongly influenced by copolymer composition and block architecture [23–25].

Overall, DSC thermograms showed no substantial changes following VH₂O₂ sterilization, indicating that the thermal characteristics established during electrospinning were preserved across all polymer systems.

### ATR-FTIR Analysis

**Fig. 4:**
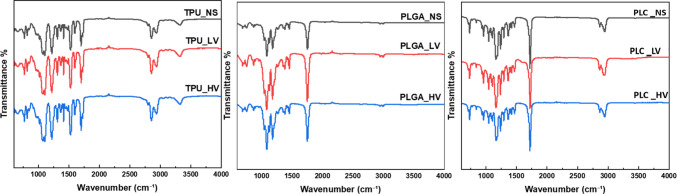
ATR-FTIR measurements of electrospun mats before (NS) and after sterilization at LV or HV conditions.

ATR-FTIR characterization was performed on electrospun scaffolds to compare non-sterile and VH₂O₂ -sterilized samples under LV and HV conditions, assessing potential bond disruptions or chemical alterations induced by the sterilization process (Fig. 4). The analysis showed no variations in chemical composition and confirmed bond integrity. For PLC, characteristic peaks included C = O stretching at 1743 cm⁻^1^, C–O–C stretching at 1084, 1128, and 1181 cm⁻^1^, and broad -OH stretching between 3200–3600 cm⁻^1^, along with C-H and CH₃ signals at 2993 and 1045 cm⁻^1^ [26]. PLGA exhibited OH group absorbance between 3450–3500 cm⁻^1^, C-H stretching from 2885–3010 cm⁻^1^, C = O stretching at 1762.6 cm⁻^1^, and C-O stretching between 1186–1089 cm⁻^1^. Consistent as cited literature TPU spectra showed NH stretching between 3324–3307 cm⁻^1^, CH₂ bending at 1435 cm⁻^1^, and C–O–C stretching at 997 cm⁻^1^ [27–29]. Comparative analysis revealed overlapping spectral features across PLC, PLGA, and TPU, supporting a consistent interpretation of chemical structure across materials and conditions. The presence of C-H stretching vibrations in aliphatic hydrocarbon chains and C = O stretching vibrations of esters indicated structural similarities and shared functional groups among these polymers.

### Morphological Analysis and Surface Wettability

**Fig. 5:**
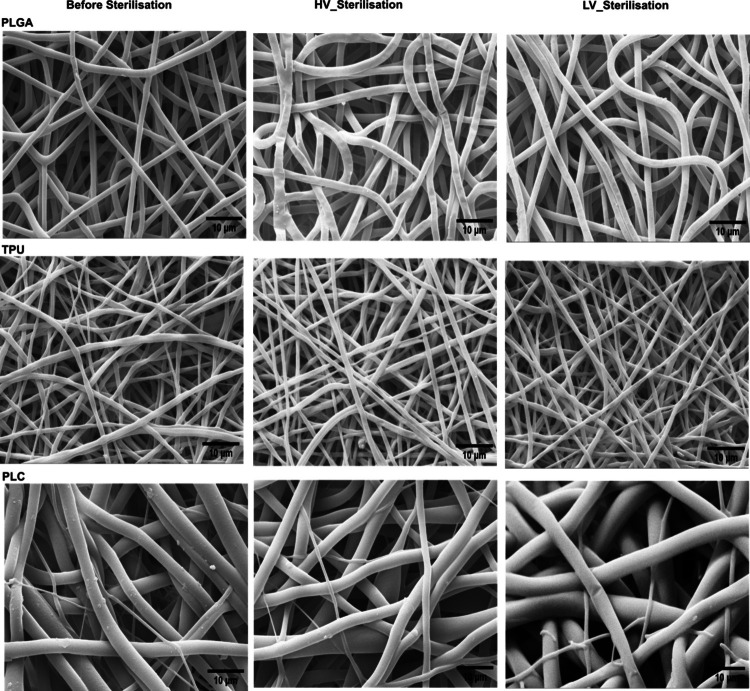
SEM images of electrospun mats fabricated from medical-grade TPU, PLGA, and PLC following sterilization under HV and LV conditions. Images illustrate the morphological effects of vacuum level on fiber structure, highlighting fiber compression, slightly increased diameter, and pore area reduction, particularly in PLGA (*Magnification 5.04 K X*).

SEM and contact angle measurements were performed to evaluate morphological and surface property changes in electrospun mats made from TPU, PLGA, and PLC. For each polymer, mats were electrospun in single batches and divided into three matched groups: non-sterilized (NS), sterilized under LV, and sterilized under HV using VH₂O₂. This design allowed for paired comparisons, with statistical evaluation performed using paired t-tests (*n* = 50 fibers per group). Fiber diameters and porosity were quantified using Image J plugins [30]. SEM analysis **(**Fig. 5**)** revealed that HV sterilization led to a slight fiber compression across all materials.

A different behavior can be observed for fibers made of different materials. TPU and PCL matrices show an increase in fibers diameter when sterilized under HV conditions, accompanied by a substantial decrease in porosity (approximately one-half and one-third, respectively, although these variations may not be particularly significant due to the high standard deviation). Conversely, LV sterilization did not substantially induce dimensional changes in the TPU and PCL fibers, while only the PLC scaffolds showed an apparent reduction in porosity. PLGA mats display a more pronounced increase in fiber size when sterilized under LV conditions, where a reduction in matrix surface porosity is also observed.

**Fig. 6:**
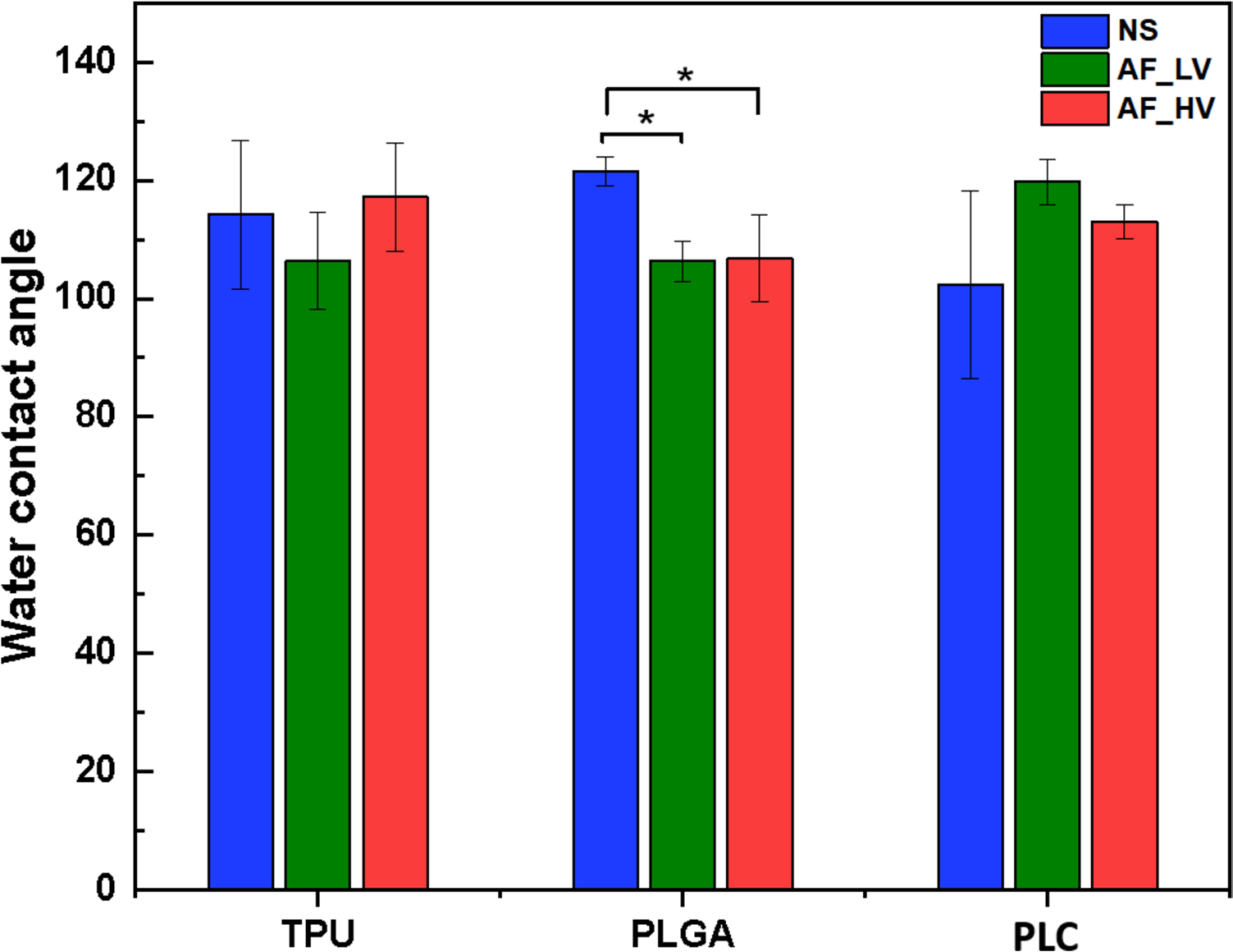
Water Contact angle measurement of non-sterilized electrospun mats (NS) and mats after sterilization at LV (AF_LV) and HV conditions (AF_HV). Data are presented as mean ± SD (*n* = 5). For PLGA, both AF_LV and AF_HV groups show significantly lower contact angles compared to NS scaffolds (p < 0.05, one-way ANOVA with post-hoc comparison), whereas no statistically significant differences were observed for TPU or PLC scaffolds.

Contact angle measurements (Fig. 6) showed that only PLGA exhibited a significant change in surface wettability following both HV and LV sterilization, reflected by a measurable increase in hydrophilicity with respect to non-sterilized mats. This observation is supported by strong statistical evidence, with highly significant differences among PLGA conditions (ANOVA *p* = 1.61 × 10⁻^5^; Kruskal–Wallis *p* = 7.3 × 10⁻^4^). In contrast, TPU and PLC displayed no statistically significant changes in contact angle across vacuum levels, consistent with stable surface properties. A significance threshold of *p* < 0.05 was applied for all analyses.

The estimated surface porosity provides a two-dimensional surface pore area fraction rather than true volumetric porosity. Although porosity is inherently a 3D property, surface measurements can still reflect morphological changes. VH₂O₂ sterilization indifferent vacuum conditions led to increased fiber diameter and reduced surface pore area (Table II), indicating fiber flattening and partial structural collapse. While such compression typically reduces surface roughness and may enhance hydrophobicity, PLGA instead showed increased wettability (i.e., decreased contact angle; Fig. 4). This apparent contradiction can be explained by a shift in wetting behavior: fiber flattening likely disrupted microscale air pockets supporting the Cassie–Baxter regime, favoring a Wenzel-type wetting in which liquid penetrates the surface more easily [31, 32]. FTIR analysis confirmed no chemical changes, suggesting that the enhanced wettability in PLGA results from physical surface reorganization rather than chemical modification or surface energy changes.

**Table II:** Comparison of Mean Diameters and Average Pore Areas for TPU, PLGA and PCL electrospun scaffolds, non sterilized (_NS) or after sterilization in HV (_HV) or LV (_LV) conditions (count of fibers *n* = 50)

Sample Name	Mean Diameter (µm)	Average Pore area (µm^2^)
TPU_NS	0.92 ± 0.42	1.56 ± 1.43
TPU_HV	1.14 ± 0.27	0.57 ± 0.72
TPU_LV	0.96 ± 0.26	1.54 ± 1.78
PLGA_NS	1.55 ± 0.21	1.65 ± 1.91
PLGA_HV	1.77 ± 0.30	1.32 ± 1.54
PLGA_LV	1.89 ± 0.50	0.61 ± 1.25
PLC_NS	2.65 ± 1.30	5.48 ± 5.09
PLC_HV	2.89 ± 1.10	2.29 ± 1.17
PLC_LV	2.38 ± 1.30	3.30 ± 2.93

### Mechanical Characterization

Mechanical testing results (Table III) showed that neither LV nor HV VH₂O₂ sterilization significantly affected the mechanical properties of TPU, PLC, or PLGA electrospun mats (p > 0.05, *n* = 3). TPU and PLC retained low Young’s modulus (< 1 MPa) and high elongation at break (> 70%) after sterilization, consistent with their elastomeric behavior [28, 33, 34]*.* PLGA mats exhibited higher stiffness (Young’s modulus ~ 19–26 MPa) and lower elongation (~ 20–30%), with no significant changes in UTS or modulus following sterilization. A slight reduction in PLGA elongation was observed, but this was not statistically significant (p > 0.05).

**Table III:** Mechanical property of TPU, PLC and PLGA electrospun mats before (NS) and after sterilization in different vacuum conditions (*n* = 3)

Polymer	*Vacuum Condition	UTS (MPa)	Young’s Modulus (MPa)	Yield Strength (MPa)	Elongation at Break (%)
TPU	LV	12.7 ± 1.8	1.0 ± 0.4	-	86 ± 17
TPU	HV	14.7 ± 3.2	1.0 ± 0.2	-	90 ± 6
TPU	NS	9.2 ± 3.3	0.8 ± 0.3	-	76 ± 12
PLC	LV	3.7 ± 1.0	0.35 ± 0.08	-	57 ± 1
PLC	HV	2.4 ± 0.6	0.22 ± 0.04	-	61 ± 2
PLC	NS	3.5 ± 2.3	0.29 ± 0.17	-	68 ± 8
PLGA	LV	3.2 ± 0.7	19.5 ± 0.6	3.6 ± 0.5	23 ± 3
PLGA	HV	4.3 ± 0.9	19.1 ± 2.6	4.2 ± 0.5	20 ± 9
PLGA	NS	5.3 ± 2.1	26 ± 1.6	4.8 ± 1.9	30 ± 3

*Sterilised in Low Vacuum (LV = 10 mbar), Sterilised in High vacuum (HV = 2 mbar), Non-Sterilised (NS)

Although PLGA fiber diameter increased slightly after sterilization (Table II), mechanical performance remained unchanged. In contrast to gamma irradiation and ethylene oxide, which have been reported to reduce molecular weight and induce structural shrinkage in PLGA mats, VH₂O₂ sterilization preserved mechanical integrity in this study [33, 35].

## Conclusion

In this work, we focused specifically on medical grade polymers potentially useful as components of hybrid vascular graft systems as well as other short-term barriers, guided-tissue-regeneration, and drug-delivery applications. The PLGA and PLC grades used were intentionally selected for resorbable, non-load-bearing layers designed to undergo controlled, sequential degradation to support staged tissue regeneration and drug release. TPU, in contrast, serves as the biocompatible structural layer, providing the long-term mechanical strength, elasticity, and compliance required to match native vascular tissue.

This study demonstrates that LV VH₂O₂ sterilization is an effective and polymer-friendly method for sterilizing medical-grade PLGA-, PLC-, and TPU-based electrospun scaffolds, without compromising molecular integrity or chemical stability. While the sterilization response was material-dependent, with electrospun PLGA exhibiting modest fiber compression and increased wettability — particularly under prolonged LV exposure — these changes were limited and did not impair scaffold functionality. The observed increase in fibers diameter is attributed to the rupture of surface air pockets, consistent with the Wenzel wetting model. Notably, raw polymers and PLC scaffolds remained stable even under room-temperature sterilization, highlighting the feasibility of energy-efficient processing.

While further validation across diverse polymer systems and processing conditions is needed, additional characterization studies should be also focusing on potential changes, including morphological alterations, depending on the intended application of the final product (*i.e.* according to the medical devices’ risk classification and their intended use). Overall, these results provide a robust physicochemical assessment of sterilization effects, representing a necessary prerequisite for subsequent biological evaluation. VH₂O₂ sterilization emerges as a gentle, green, and residue-free approach that preserves scaffold morphology and performance, supporting its potential as a sustainable and versatile sterilization strategy for biomedical applications.

## Supplementary Information

Below is the link to the electronic supplementary material.

ESM 1 (DOCX 6.16 MB)

## Data Availability

The data sets generated during and/or analyzed during the current study are available from the corresponding author on reasonable request.
