# Supplementary material for: Advancing Sterilization of Medical Implant Polymers: Novel Low-Temperature Deep-vacuum Vaporized H2O2 Technology Surpasses Current Methods
Source: Pharm Res. 2026 Mar 16;43(4):1259–70. doi: 10.1007/s11095-026-04063-x (PMC13179216; doi:10.1007/s11095-026-04063-x)
Supplement: Supplementary file 1 — (DOCX 6.16 MB) [file 11095_2026_4063_MOESM1_ESM.docx]

Advancing Sterilization of Medical Implant Polymers: Novel Low-Temperature Deep-vacuum Vaporized H_2_O_2_ Technology Surpasses Current Methods

**Abinaya Nallathambi^1^, Mariella Rosalia^1^, Enrica Chiesa^1^, Giovanna Bruni^2^, Aurora Tamborini^3^, Sergio Crotti^3^, Ida Genta^1,^***

^1^Department of Drug Sciences, University of Pavia, 27100 Pavia, Italy

^2^Department of Physical Chemistry, University of Pavia, 27100, Pavia, Italy

^3^De Lama S.p.A. Process and Sterilization Solution, 27028 S. Martino Siccomario (Pavia), Italy

**Supplementary**

a)

b)

**Fig. S1** (**a**) Gel permeation chromatograms of PLGA, PLC, and TPU raw materials before (_raw) and after sterilization at different temperatures (20_, 30_, 40_, 50_S). Notably, PLC exhibited a glass transition temperature around 19 °C, and therefore no higher temperature exposure was applied. (**b**) Gel permeation chromatograms of electrospun PLGA, PLC, and TPU mats before (_NS) and after sterilization (_S) at the optimized temperatures.

a)


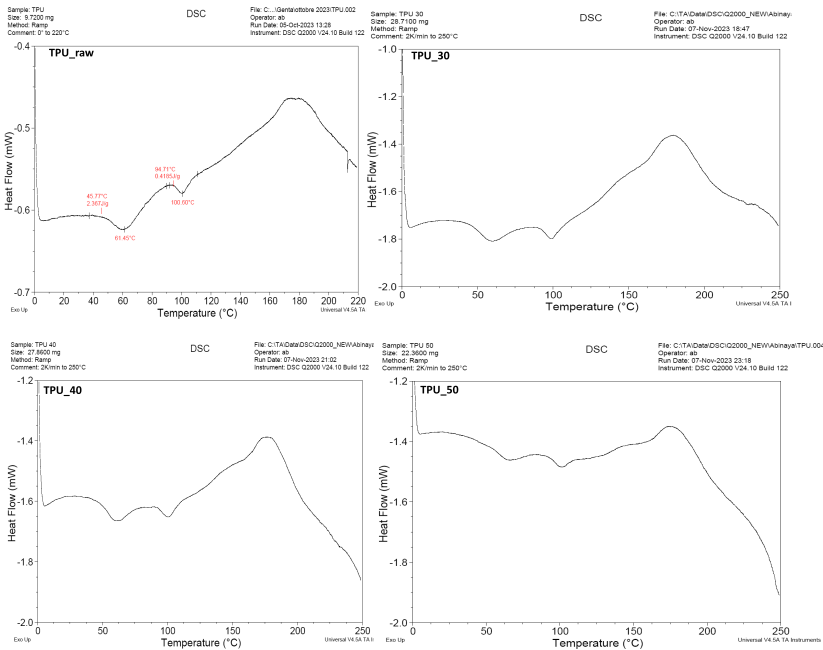


b)


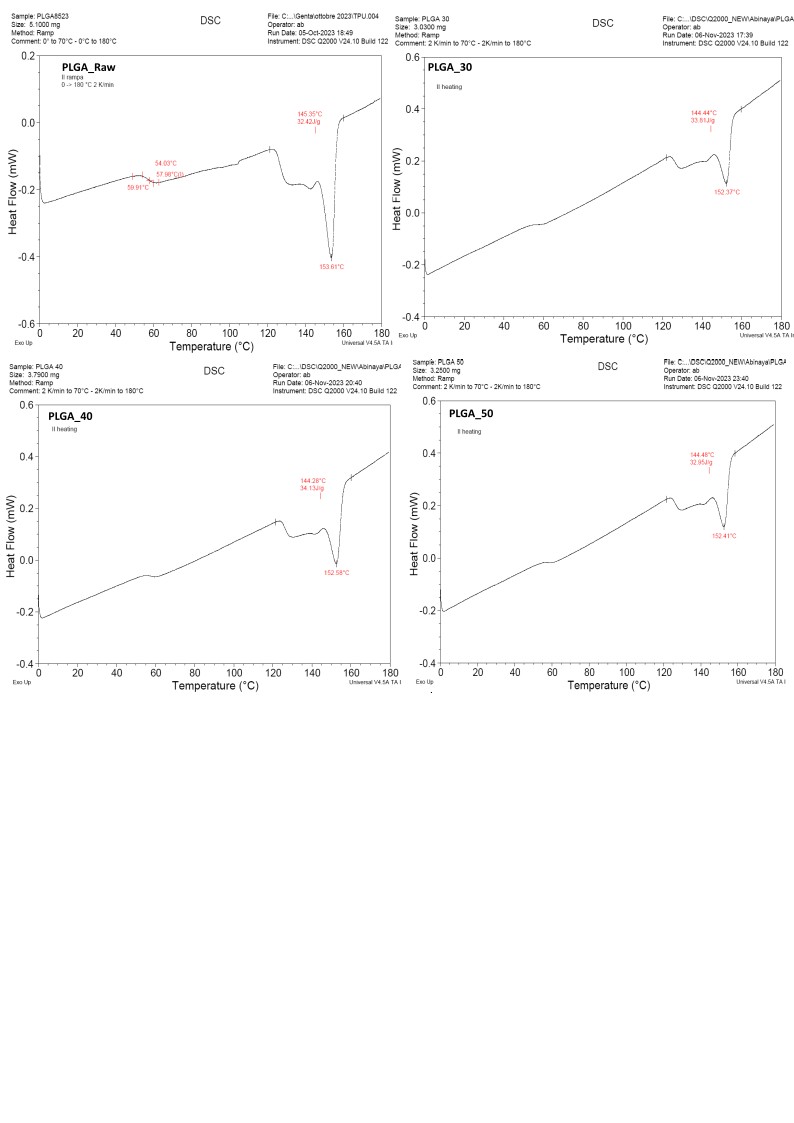
\

c)


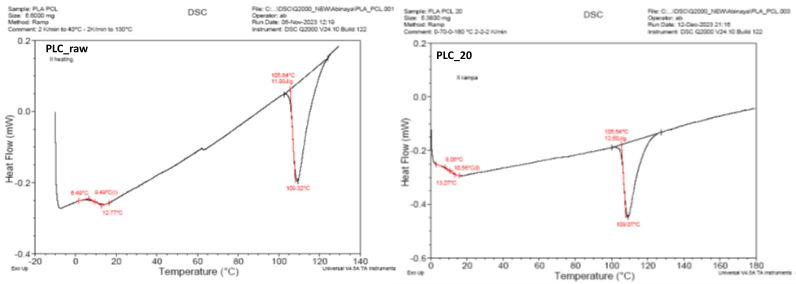


**Fig. S2** Differential scanning calorimetry (DSC) characterization of raw polymer materials before and after sterilization: (**a**) TPU, (**b**) PLGA, and (**c**) PLC. Sterilization was performed at 30, 40, and 50 °C for TPU and PLGA, at 20-25 °C for PLC, corresponding to the optimized conditions determined from preliminary DSC analysis. No significant changes in glass transition temperature (T_g_) or thermal behavior were observed after sterilization, indicating that the molecular and thermal integrity of the polymers was preserved under the applied sterilization conditions.


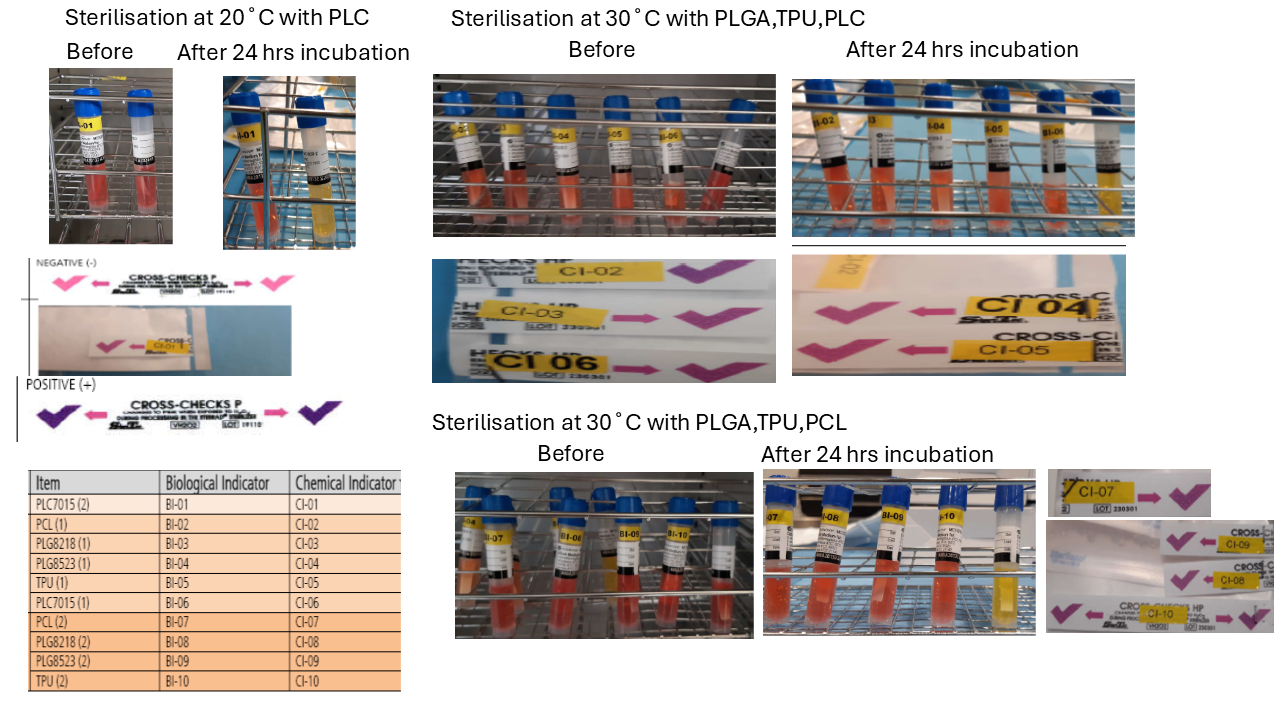


**Fig. S3** Representative sterility validation of raw polymer materials using biological indicators (BIs) and chemical indicators (CIs) under different sterilization conditions. (**Top left**) PLC samples sterilized at 20 °C under HV conditions; images show BIs before and after 24 h incubation, with color change confirming sterility. (**Top right**) PLGA, TPU, and PLC samples sterilized at 30 °C; BIs and CIs are shown before and after 24 h incubation. (**Bottom**) Sample replicate of PLGA, TPU, and PLC samples sterilized at 30 °C with corresponding BIs and CIs. Chemical indicators show color change from purple to pink upon exposure to vaporized H₂O₂, confirming sufficient sterilant penetration, while biological indicators retain red color after incubation, indicating complete spore inactivation. Negative and positive controls (CI strips and unsterilized BIs) are included to validate the testing system.

**Fig. S4** ATR-FTIR of non-sterilized raw material (_R) and electrospun mats (_Ns) based on TPU, PLC and PLGA.
